# An Interactive, Bilingual Touch Screen Program to Promote Breastfeeding Among Hispanic Rural Women: Usability Study

**DOI:** 10.2196/resprot.2872

**Published:** 2013-11-07

**Authors:** Ashish Joshi, Susan Wilhelm, Trina Aguirre, Kate Trout, Chioma Amadi

**Affiliations:** ^1^Center for Global Health and DevelopmentCollege of Public HealthUniversity of Nebraska Medical CenterOmaha, NEUnited States; ^2^College of NursingUniversity of Nebraska Medical CenterOmaha, NEUnited States

**Keywords:** usability, breastfeeding, education, evaluation, computer

## Abstract

**Background:**

Computer technology can be effectively used to educate patients and improve knowledge and attitudes, leading to healthier behavior. Among rural women, breastfeeding outcomes seem to be worst compared to women living in urban areas. The implementation of a bilingual computer mediated health education program to disseminate information and improve outcomes among users with low literacy levels has proven to be successful.

**Objective:**

The objective of this pilot study was to examine the usability of an interactive, bilingual touch screen computer-based educational program to promote breastfeeding practices among Hispanic women living in rural settings.

**Methods:**

A convenience sample of 10 Hispanic rural women at the Regional West Medical Center (RWMC), Scottsbluff was enrolled during May 2013. Information about this cross-sectional study was made available through the flyers at the RWMC. A brief introduction of the prototype was given and study subjects were then asked to complete a predefined set of tasks by interacting with the prototype. Users were assigned 6 tasks and information was gathered about the time taken to complete the tasks, number of attempts, and if assistance was needed. Notes and test sessions were audiotaped. Usability assessment was performed using the System Usability Scale (SUS).

**Results:**

The mean age of the study participants was 28 years (SD 3.6), the majority of them had 12 or more years of education (90%, 9/10), and 60% (6/10) had breastfed less than 6 months. There were 90% (9/10) of the study participants that had no prior history of taking prenatal classes and 80% (8/10) that did not intend to take any prenatal classes in the future. The average SUS scores were 90 and SD was 10.5. There were three participants that had average SUS scores of 100, followed by scores of 97.5 (1/10), 95 (1/10), 87.5 (1/10), 85 (2/10), 82.5 (1/10), and one participant had a score of 67.5 (1/10). No assistance was needed to complete any of the tasks.

**Conclusions:**

The study participants were able to navigate through the multimedia program with ease and obtain relevant breastfeeding related health information. The interactive, touch screen computer-based breastfeeding program had high acceptance among 10 Hispanic women living in rural settings.

## Introduction

### Children and Breastfeeding

Breastfeeding is essential for children to have a healthy and regular development. The American Association of Pediatrics advises women to breastfeed exclusively for the first six months of the child’s life [1]. Internationally, the World Health Organization also recommends that infants should be exclusively breastfed for the first six months of life, to achieve optimal growth, development, and health [2]. Breast milk provides the best nutritional source for the healthy growth and development of children and it is protective against a variety of diseases [3,4]. Rates of breastfeeding continue below the goals proposed by Healthy People 2020 [5,6].

Among rural women, breastfeeding outcomes seem to be worst compared to women living in urban areas [7]. Research has shown that breastfeeding practices depend on a multitude of factors including knowledge and perception of breastfeeding, socioeconomic status, cultural background, and family relationships [8,9]. Lower prevalence of breastfeeding initiation has been observed among Hispanics living in western states compared to non-Hispanic whites [10]. Many challenges exist to improve breastfeeding practices. Family and friends may provide inaccurate information about breastfeeding, and mothers often perceive social disapproval [11]. Information presented should be tailored, culturally appropriate, and relevant to improve breastfeeding related knowledge, change behaviors, and practices related to breastfeeding initiation and duration [12].

### Computer Technology and Breastfeeding

Computer technology can be effectively used to educate patients and improve knowledge and attitudes, leading to healthier behavior [13]. Prior research has shown successful interventions that facilitate behavior modification and health promotion through use of Information and Communication Technologies (ICT) [14]. Health information tailored through use of ICT has proven to be more effective and efficient [15-17]. Computer-based tailoring is a process of creating individualized communication and is an assessment-based approach in which individuals provide personal data related to a given health outcome [18]. Those data are then used to determine the most appropriate information or strategies to meet each individual’s unique need. An important theoretical basis for tailoring comes from the Elaboration Likelihood Model [19], which states that people are more likely to actively and thoughtfully process information if they perceive it to be personally relevant. Messages processed in this way tend to be retained for a longer period of time and are more likely to lead to permanent attitudinal change.

### Usability Testing

Usability testing is a key component of product evaluation, and focuses on measuring a product’s ability to meet its intended purpose by providing evidence on how real users interact with it [20]. Additional methods of usability testing include expert evaluators to be able to identify problems and determine whether the user conforms to established usability principles in a heuristic evaluation. Expert evaluation can identify errors in a systematic process, and accurately diagnose them within the system design [21]. Usability problems are important to be evaluated such as: (1) revision of instructions and functionality, (2) better introductory instructions, (3) elimination of instructional messages, and (4) simplified representation and improved labeling [21]. Several interactive health care applications often have been hampered by their poor design, making these systems difficult to learn and complicated to use [22]. The systems designed without taking into account the target users can result in poor adoption of these systems. Tailoring the design of the systems helps meet the specific needs of the users and results in increased productivity, reduced errors, reduced need of user training and user support, and improved acceptance [22]. This will allow the users to operate the system effectively rather than struggling with the computer's functions and user interface, enhancing users’ productivity [23]. The implementation of a bilingual computer mediated health education program to disseminate information and improve outcomes among users with low literacy levels has proved to be successful [24]. Usability testing is an important process in the design and implementation of programs to allow for further development and better adoption of technologies in populations. Usability testing can be conducted among target populations, and can help identify key issues before full implementation.

The purpose of this pilot study was to examine the usability of an interactive, bilingual, touch screen enabled standalone and Internet based breastfeeding educational program among Hispanic women living in rural settings and in this case Scottsbluff, Nebraska.

## Methods

### The Convenience Sample

We enrolled a convenience sample of 10 Hispanic rural women at the Regional West Medical Center (RWMC), Scottsbluff, Nebraska during May 2013. Information about this cross-sectional study was made available through the flyers at the RWMC. The optimum number of participants for in-depth interviews is typically 6-10 people with similar backgrounds who participate in the interview for one to two hours, however interview times may vary [25]. Prior literature has suggested that smaller groups of a sample between 6 to 10 participants show greater potential and large enough to gain a variety of perspectives, and small enough not to become disorderly or fragmented [25]. After a brief introduction to the system, study subjects were asked to complete a predefined set of tasks by interacting with the system and the duration of this interaction was around 30 minutes. The study participants used a think aloud protocol involving participants thinking aloud as they used the program, and were asked to say whatever they were looking at, thinking, doing, and feeling. Then, users were assigned 6 tasks including: (1) entering age, (2) moving forward to the next question, (3) ability to pause the program, (4) replay, (5) use of help module, and (6) ability to change the settings. Tasks were chosen based on varied complexity. Information was gathered about the time taken to complete the task, task completion (yes or no), number of attempts taken to complete the task, and whether or not assistance was sought during the completion of the task. Notes and test sessions were audiotaped of everything that users said. Usability scores were recorded among the 10 study participants, where vast amounts of usability problems and issues can be identified with only a small number of test subjects, as few as 8 to 10 participants [26]. The UNMC Institutional Review Board (IRB protocol#430-12-EP) approved the study.

### Variables Description

#### Sociodemographics

Information gathered included age (years), years of education, income, marital status, and employment status. Information was also gathered about the history of previous pregnancies, duration of breastfeeding, prior history of prenatal breastfeeding classes, or any intend to take these classes in the future. Information was also gathered about the sources of health information utilized by the study participants. Further information gathered included prior use of computers and Internet and the frequency of use.

#### Think Aloud Analysis

Users were asked to navigate through the program when they were assigned tasks. Qualitative data were generated as they were asked to say whatever they were looking at, thinking, doing, and feeling as they go about their task, enabling observers to see first-hand the process of task completion. Audios were recorded and notes were taken.

#### Task Assessments

The study participants were assigned 6 tasks to complete and information recorded included the task completion time, number of attempts an individual made to complete the task, and whether or not assistance was taken to complete that task.

The tasks chosen are the most common tasks that users would be using to interact with the system. Tasks were chosen based on varied levels of complexity. Notes were taken and audios were recorded.

#### System Usability Scale

A Likert scale survey System Usability Scale (SUS) was used to assess user acceptance with an interactive, computer-based breastfeeding educational program and any recommendations that the study subjects might have to improve the system. SUS is a 10-item questionnaire with 5 response options ranging from strong agreement to strong disagreement. Possible scores are 0, 1, 2, 3, or 4 for each question. To minimize bias based on agreement or disagreement, odd items of the SUS questionnaire are given more points for strong agreement, and even numbered items are given more points for strong disagreement. The total score is calculated from adding up the converted responses for each user and multiplying that total by 2.5. This converts the range of possible values from 0 to 100 instead of from 0 to 40.

#### User Acceptance

We adapted and used previously published questionnaires used to assess acceptance of a computer-based asthma educational program [27]. Feedback was gathered on ease of use of program, navigation patterns, future use of the program, and if others would be recommended to use the program.

#### Statistical Analysis

Descriptive analysis was performed to report means and standard deviations of the continuous variables and frequency distribution of the categorical variables. Analysis of the qualitative data was performed on the data collected through written notes and recorded spoken language. A thematic approach was used from the narratives of research participants gathered during the think aloud approach to identify themes or patterns, organize data into coherent categories, and then interpret the findings of the data. Qualitative data were analyzed based on grounded theory. All quantitative analysis was performed using SAS version 9.1.

### Patient Education and Motivation Tool

#### The Patient Education and Motivation Tool Program

Patient Education and Motivation Tool (PEMT) is a touch screen computer-based interactive health education program designed integrating a variety of cognitive-behavioral theories. PEMT facilitates health information and messages to be adapted depending on the psychosocial elements including attitude, self-efficacy, expectations, personal norms, and social influences (Table 1). PEMT has 3 key components. Screening is the first component, which gathers individual sociodemographics, knowledge, attitudes, and practice through a series of questions. Second, the learning component delivers educational material in a structured format. The entire educational material is broken down into a series of modules, each module into submodules and each submodule into a series of educational messages. Each message is then presented using various multimedia formats (such as audio, video, text, and images. Third, the evaluation component gauges effectiveness of the program by assessing change in knowledge, attitudes, and practices of the individuals. The main objective of the PEMT is to present health information in an interactive tailored manner considering multiple factors influencing health status and health behaviors.

**Figure 1:**
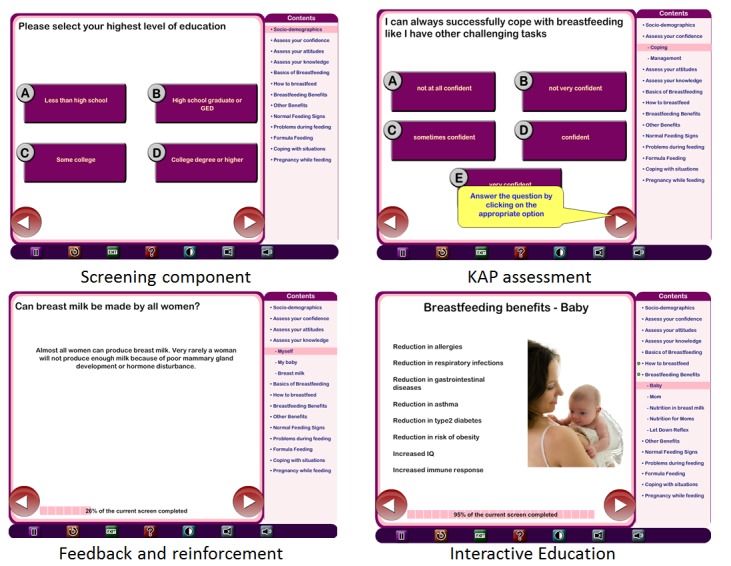
Components of computer based education program.

The existing PEMT was modified to develop an interactive, tailored, computer-based breastfeeding educational support program to educate Hispanic women living in rural settings. The computer-based Breastfeeding Educational Support program aims to provide modular, culturally relevant, bilingual (English/Spanish) breastfeeding education tailored to the needs of the mothers. Content tailoring was performed based on the needs of the Hispanic women living in rural settings and guided the development of the breastfeeding educational modules. The modules of the finalized breastfeeding educational content were made available both in Spanish and English so that the study participant can use either language to navigate through the program. The modules included: (1) basics of breastfeeding, (2) how to breastfeed?, (3) benefits of breastfeeding to mother and child, (4) normal feeding signs, (5) problems during breastfeeding, (6) formula feeding, (7) coping with breastfeeding, and (8) ability to get pregnant while breastfeeding. The entire finalized breastfeeding educational content will be broken down into a series of modules, each module into submodules and each submodule into a series of educational messages. The computer-based program will have the ability to deliver breastfeeding education in varied learning styles such as text-only, audio and text, or text, audio, and images to account for health literacy of the individuals. The program will simplify the design and create multiple tailored versions of printed materials instead of using a single standardized version. Figure 1 shows the components of the computer-based education program.

**Table 1:** Modify PEMT to adapt to computer mediated breastfeeding educational program.

Theory	Purpose	PEMT learning component
Information processing theory [28]	Present information as a meaningful unit and limited to 5-9 pieces of information	Each slide includes limited educational content
Constructivist theory [29]	Present information in a structured format simple to understand	The content presented on each screen is in a series of short messages
Cognitive flexibility theory [30]	Information presented should be highly interconnected and relevant to the learner	The educational material is related to each other
Cognitive flexibility theory [30]	Multiple content formats	Content is available as audio, images, and text
Cognitive load theory [31]	Minimize working memory load	Information is presented as a series of short educational messages
Behavioral theory and Operant conditioning [32]	Feedback given based on responses and positive reinforcement for healthy behaviors	Based on individual correct or incorrect response feedback is provided

#### PEMT Tailoring Algorithm

**Figure 2:**
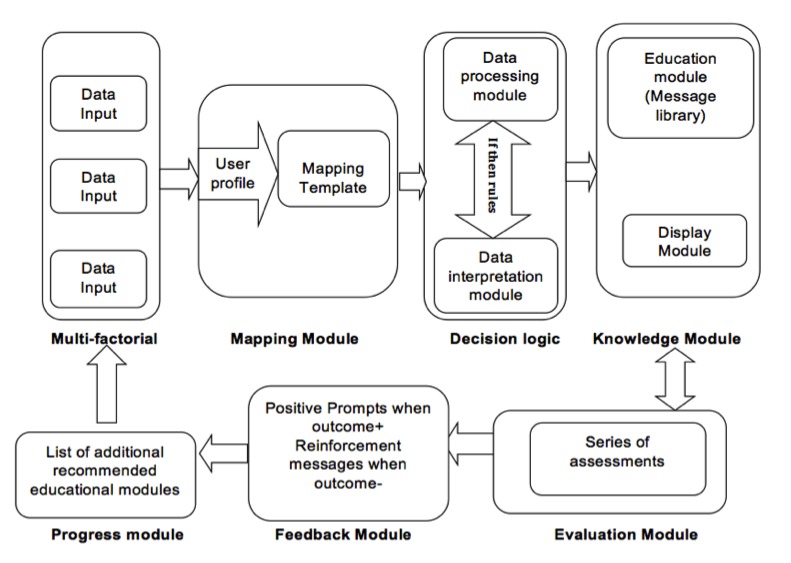
PEMT tailoring algorithm.

The PEMT tailoring algorithm involves several components including: (1) multifactor assessment that helps to gather information about several variables, (2) mapping module involves generating individual user profile, (3) decision logic involving data processing and interpretation, (4) knowledge module involves having library of educational messages and various formats in which these can be delivered, (5) evaluation module involves a series of assessments, (6) feedback module involves reinforcement based on the responses, and (7) progress module involves the topics covered (Figure 2 shows this tailoring algorithm). For example, sociodemographics and breastfeeding knowledge and attitude towards breastfeeding practices are gathered through a series of questions. For example, if a person had poor breastfeeding knowledge on certain topics; educational messages were tailored to address those gaps in the knowledge. Messages were also tailored for participants with poor breastfeeding skills and appropriate feedback was provided through reinforcement and use of motivational prompts. The program aims to deliver specific messages as computers provide a medium to adopt tailoring with no time lag and multimedia capabilities.

## Results

### The Participants and Their Sociodemographic Characteristics

Results showed the mean age of the study participants to be 28 years (SD 3.6), the majority of them had 12 or more years of education (90%, 9/10), half of them were married (50%, 5/10), 60% (6/10) had breastfed less than 6 months. The majority of the participants received a low income with one (10%, 1/10) participant’s annual incomes less than $10,000, one (10%, 1/10) between $10,000-19,000, two (20%, 2/10) between $20,000-29,000, two (20%, 2/10) between $30,000-39,000, three (30%, 3/10) between $40,000-49,000, and one (10%, 1/10) was greater than $50,000. The majority of the study participants had either no prior history of taking prenatal classes (90%, 9/10) or did not intend to take any prenatal classes in the future (80%, 8/10) (Table 2).

**Table 2:** Study population characteristics (N=10).

Variables		Responses n (%) or mean (SD; range)
Age, years		28.0 (3.6; 23-35)
Education ≥12 years		9 (90)
Marital status, married		5 (50)
Employment, full time		8 (80)
Income status, $40,000-49,000		3 (30)
Smoking status, never smoked		9 (90)
# of children, mean (SD; range)		2 (1; 0-4)
Previous history of pregnancies		2 (1; 0-4)
**Breastfeeding history**		
	Breastfeeding duration	None=3; 1-5 months=4; 6-12months=1; >12months=2
	Number of children breastfed	1.44 (1; 0-3)
	Previous history of taking prenatal classes; no	9 (90)
	Intent to take prenatal classes in the future; no	8 (80)

### Familiarity With Use of Technology and Internet Enabled Breastfeeding Information

#### Participants and Computers

**Figure 3:**
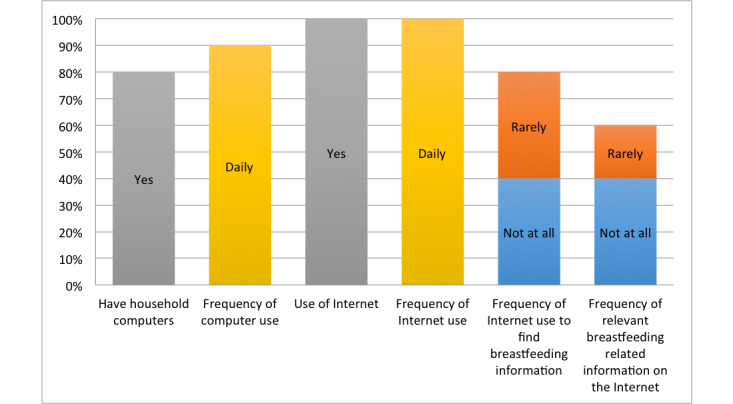
Familiarity with the use of technology and breastfeeding information.

Results showed that the majority of the study participants had household computers (80%, 8/10) and all of them were using the Internet (100%, 10/10). The frequency of using computers daily was 90% (9/10) compared to 10% (1/10) who used computers once a week. The frequency of using the Internet daily was 100% (10/10). More than half of them were using the Internet to find breastfeeding related information (60%, 6/10) compared to the 40% (4/10) that never used the Internet. Of those who used the Internet, 40% (4/10) used it rarely, compared to those who either used it once a week (10%, 1/10) or once a month (10%, 1/10). Only 20% (2/10) of the study participants agreed that they always found relevant breastfeeding information on the Internet compared to the other 20% (2/10) who would sometimes find relevant information related to breastfeeding. There were 20% (2/10) of the study participants that rarely found relevant breastfeeding information on the Internet (Figure 3 shows familiarity with the use of technology and breastfeeding information).

#### Task Assessments

There were 6 tasks assigned to each study participant. One hundred percent of the study participants did not need assistance to complete any of the tasks. The average total time taken to complete all the tasks was 17.4 seconds (SD 6.39). More time was taken to complete certain tasks (Table 3). There were three tasks that were completed during the first attempt, compared to the others that were completed during the second attempt. No assistance was needed to complete any of the tasks.

#### Thematic Analysis Using Think Aloud Data

One hundred percent of the study participants agreed that the educational content enhanced with visual images was sufficient to meet their informational needs related to breastfeeding. However, one participant suggested of “having more information about milk storage and pumping breast milk.” Two participants agreed about having additional pictures and less text to enhance their understanding about the breastfeeding related information “I am a visual person, it helps me understand better.”

All study participants found this interactive, computer-based breastfeeding educational program easy to navigate (100%, 10/10). The majority of the participants found the program “self-explanatory, easy to figure out, and simplified.” Some of the participants thought that a drop-down menu function would be good to gather information about various variables (40%, 4/10). There were 30% (3/10) of the participants that thought that the ability to customize colors based on the gender of the baby would be useful. The various functions of the program including the play/pause button, audio, and images were extremely beneficial. The help function was very useful and one of the participants felt that “help section is informative to let me know how to proceed to the next section.”

The labeling of buttons, highlighting keywords, videos, able to make distinctions between two screens, and the ability to self select the choice of medium to acquire breastfeeding related information (audio or text) could be additional features that can be added to the existing program. There were two study participants that also felt that a progress monitor and a summary report at the end of the program would be useful to help them show how far they have to complete the tasks and how much more they still have to go.

#### Usability of the Program

The average SUS scores were 90 and SD was 10.5. There were three participants that had average SUS scores of 100, followed by scores of 97.5 (1/10), 95 (1/10), 87.5 (1/10), 85 (2/10), 82.5 (1/10), and one participant had a score of 67.5 (1/10) (Table 4).

**Table 3:** Task completion times, attempts, and assistance needed to complete the tasks (N=10).

Task types	Task completion time, mean; (SD) range	Tasks completion during the 1^st^ attempt, n (%)	No assistance needed, n (%)
T1 select age	3.94; (1.42) 2.30-6.80	10 (100)	10 (100)
T2 move to next slide	2.11; (2.4) 0.5-8.50	9 (90)	10 (100)
T3 pause program	2.48; (1.31) 0.5-5.30	10 (100)	10 (100)
T4 replay	2.23; (1.38) 0.9-5.80	7 (70)	10 (100)
T5 using help	2.75; (1.96) 1.0-7.50	10 (100)	10 (100)
T6 change settings	3.89; (5.55) 0.8-19.40	9 (90)	10 (100)

**Table 4:** Frequency distribution of the SUS.

Variables	Frequency, n (%)				
	1 Strongly disagree	2	3	4	5 Strongly agree
I think that I would like to use this system frequently.			1 (10)	3 (30)	6 (60)
I found the system unnecessarily complex.	10 (100)				
I thought the system was easy to use.				1 (10)	9 (90)
I think that I would need the support of a technical person to be able to use this system.	10 (100)				
I found the various functions in this system were well integrated.				4 (40)	6 (60)
I thought there was too much inconsistency in this system.	9 (90)				1 (10)
I would imagine that most people would learn to use this system very quickly.	1 (10)			2 (20)	7 (70)
I found the system very cumbersome to use.	5 (50)	1 (10)	1 (10)		3 (30)
I felt very confident using the system.	1 (10)			1 (10)	8 (80)
I needed to learn a lot of things before I could get going with this system.	10 (100)				

### High Program Acceptance Rate

**Figure 4:**
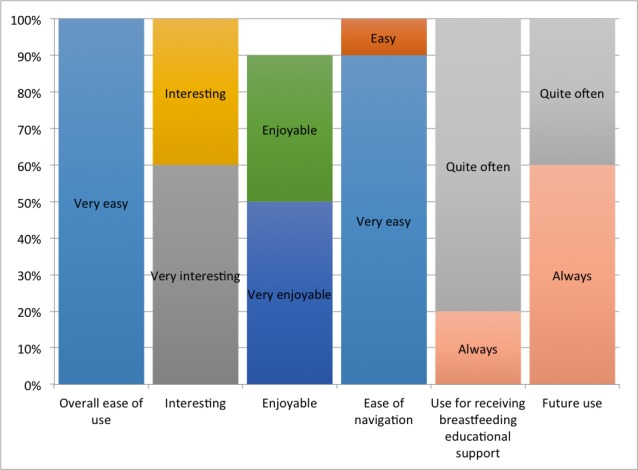
Frequency distribution of the program acceptance.

Overall, the interactive, touch screen computer-based breastfeeding program had high acceptance. There were 100% (10/10) of the study participants that found the program very easy to use, 60% (6/10) of them found it very interesting, and more than half of them (80%, 8/10) would use it quite often for receiving breastfeeding educational support. More than half of them would always want to use the program in the future (60%, 6/10). Figure 4 shows the distribution of the program acceptance.

## Discussion

### Promoting Breastfeeding

Despite the clear benefits of breastfeeding to mother and infant, breastfeeding rates continue to remain below the recommendation levels in the United States, most notably among low income mothers living in rural settings. Modifiable factors such as maternal attitudes and self-efficacy demonstrate a positive relationship with continued breastfeeding. The promotion of breastfeeding is of utmost importance because of its role in many health related outcomes. There are many challenges that prevent Hispanic women from breastfeeding, these include embarrassment, pain, inconvenience, lack of breastfeeding support, and not being able to consume alcohol or smoke cigarettes [33,34]. These barriers need to be addressed in a culturally sensitive manner that focuses on how women can overcome them. The delivery of health information is challenging for non-English speaking populations due to well-known language, low literacy, and cultural barriers.

### SUS Scores

Research has translated the average SUS score to be easily interpreted by others where products that scored in the 90s are exceptional, products that score in the 80s are good, and products that score in the 70s were acceptable, and below 70 is concerned to have usability issues that cause concerns [35]. Results of this study among 10 low-income Hispanic women living in a rural setting suggest that an interactive, computer-based breastfeeding educational program is highly acceptable with an average SUS score of 90. The interactive program included a series of breastfeeding educational modules in English and Spanish. Each module was broken down into submodules and each submodule was further broken down into a series of short educational messages. The educational messages were enhanced using various formats of multimedia in the form of text, audio, and images.

### Participants’ Computer Program Results

The majority of the participants had familiarity with the use of computers and had access to the Internet, however only 60% (6/10) of them used the Internet to find breastfeeding related information. Most of the participants did not find the breastfeeding related information on the Internet useful. Results of our study showed that all the participants were able to complete all the tasks unassisted during the use of the program. All the participants agreed that use of images and audio enhanced their understanding about the various breastfeeding educational modules. However, study participants believed that labeling of the buttons, highlighting keywords, and use of videos can further make the program interactive and useful. The system demonstrated high usability scores, reflecting its usefulness among rural Hispanic women. The usability testing of the program has helped to take into account the preferences of the target group*.*

### Health Information Technology Usability

Prior research suggests that technology is a promising way to change a person’s health behavior. Usability factors are a major obstacle to health information technology (IT) adoption. Lack of attention to health IT evaluation may result in an inability to achieve system efficiency, effectiveness, and satisfaction. Consequences may include frustrated users, decreased efficiency coupled with increased cost, disruptions in workﬂow, and increases in health care errors [36]. It is essential to be attentive to health IT usability, keeping in mind its intended users, task to be performed, and environment. Further, the usability evaluation should not only be done through use of questionnaires that provide subjective information, but should also include objective assessments through task analysis. A longitudinal study is needed to explore the acceptance of the program after the program is used for a longer duration. Future studies are planned to determine the usability among a large longitudinal study. A longitudinal study will help identify further usability challenges and program acceptance among populations with varied characteristics.

Results of our study showed high acceptance with no assistance needed by the 10 Hispanic women living in rural settings. The study participants were able to navigate through the program with ease and obtain relevant breastfeeding related health information specific to meet their needs. An interactive, touch screen breastfeeding educational program using multimedia can help overcome these barriers by delivering health education among Hispanic rural women. The results add to the growing literature demonstrating the use of touch screen technology for health education in Hispanic populations living in rural settings. Delivering interactive breastfeeding educational material in a culturally relevant manner can help facilitate change in the knowledge, attitude, and practices related to breastfeeding.

## Abbreviations

ICT: Information and Communication Technologies
IT: information technology
PEMT: Patient Education and Motivation Tool
RWMC: Regional West Medical Center
SUS: System Usability Scale

## References

[1] Gartner LM, Morton J, Lawrence RA, Naylor AJ, O'Hare D, Schanler RJ, Eidelman AI, American Academy of Pediatrics Section on Breastfeeding (2005). Breastfeeding and the use of human milk. Pediatrics.

[2] World Health Organization, World Health Organization (2003). Global strategy for infant and young child feeding.

[3] Clark SGJ, Bungum TJ (2003). The benefits of breastfeeding: An introduction for health educators. Californian J Health Promot.

[4] Hale R (2007). Infant nutrition and the benefits of breastfeeding. British Journal of Midwifery.

[5] United States Department of Health and Human Services MICH-21.

[6] Centers for Disease Control and Prevention Division of Nutrition, Physical Activity,Obesity.

[7] Flower KB, Willoughby M, Cadigan RJ, Perrin EM, Randolph G, Family Life Project Investigative Team (2008). Understanding breastfeeding initiation and continuation in rural communities: A combined qualitative/quantitative approach. Matern Child Health J.

[8] Kloeblen-Tarver AS, Thompson NJ, Miner KR (2002). Intent to breast-feed: the impact of attitudes, norms, parity, and experience. Am J Health Behav.

[9] Wells KJ, Thompson NJ, Kloeblen-Tarver AS (2002). Intrinsic and extrinsic motivation and intention to breast-feed. Am J Health Behav.

[10] Centers for Disease Control and Prevention (CDC) (2010). Racial and ethnic differences in breastfeeding initiation and duration, by state - National Immunization Survey, United States, 2004-2008. MMWR Morb Mortal Wkly Rep.

[11] Wilhelm SL, Rodehorst TK, Stepans MB, Hertzog M, Berens C (2008). Influence of intention and self-efficacy levels on duration of breastfeeding for Midwest rural mothers. Appl Nurs Res.

[12] Chapman DJ, Pérez-Escamilla R (2012). Breastfeeding among minority women: moving from risk factors to interventions. Adv Nutr.

[13] Lewis D (1999). Computer-based approaches to patient education: a review of the literature. Journal of the American Medical Informatics Association.

[14] Fogg BJ (2002). Persuasive Technology: Using Computers to Change What We Think and Do.

[15] Jones R, Pearson J, McGregor S, Cawsey AJ, Barrett A, Craig N, Atkinson JM, Gilmour WH, McEwen J (1999). Randomised trial of personalised computer-based information for cancer patients. BMJ.

[16] Kreuter M (2000). Customizing communication with computer technology. Tailoring health messages.

[17] Kreuter MW, Wray RJ (2003). Tailored and targeted health communication: strategies for enhancing information relevance. Am J Health Behav.

[18] McDonald EM, Solomon B, Shields W, Serwint JR, Jacobsen H, Weaver NL, Kreuter M, Gielen AC (2005). Evaluation of kiosk-based tailoring to promote household safety behaviors in an urban pediatric primary care practice. Patient Educ Couns.

[19] Petty RE, Cacioppo JT (1996). Attitudes and persuasion: classic and contemporary approaches.

[20] Nielsen J (1993). Usability engineering.

[21] Turner-Bowker DM, Saris-Baglama RN, Smith KJ, DeRosa MA, Paulsen CA, Hogue SJ (2011). Heuristic evaluation and usability testing of a computerized patient-reported outcomes survey for headache sufferers. Telemed J E Health.

[22] Jaspers MW (2009). A comparison of usability methods for testing interactive health technologies: methodological aspects and empirical evidence. Int J Med Inform.

[23] Maguire M (2001). Methods to support human-centred design. International Journal of Human-Computer Studies.

[24] Joshi A, Lichenstein R, King J, Arora M, Khan S (2009). Evaluation of a computer-based patient education and motivation tool on knowledge, attitudes and practice towards influenza vaccination. IEJHE.

[25] Krueger RA, Casey MA (2008). Focus groups: A practical guide for applied research.

[26] Kushniruk A, Patel VL, Cimino J (1997). Usability testing in medical informatics: cognitive approaches to evaluation of information systems and user interfaces. Proc AMIA Annu Fall Symp.

[27] Joshi A, Lichenstein R, Rafei K, Bakar A, Arora M (2007). A pilot study to evaluate self initiated computer patient education in children with acute asthma in pediatric emergency department. Technol Health Care.

[28] Miller GA (1956). The magical number seven, plus or minus two: some limits on our capacity for processing information. Psychological Review.

[29] Duffy T, Jonassen DH (1992). Constructivism and the technology of instruction: a conversation.

[30] Spiro R (1988). The Library of the University of Illinois-Urbana Champaign.

[31] Sweller J (1988). Online library Wiley.

[32] Boeree C (1998). Personality theories.

[33] Gill S, Reifsnider E, Mann AR, Villarreal P, Tinkle MB (2004). Assessing infant breastfeeding beliefs among low-income mexican americans. J Perinat Educ.

[34] Wood S, Sasonoff KM, Beal JA (1998). What's happening. Breast-feeding attitudes and practices of latino women: a descriptive study. J Amer Acad Nurse Practitioners.

[35] Bangor A, Kortum PT, Miller JT (2008). An empirical evaluation of the system usability scale. International Journal of Human-Computer Interaction.

[36] Yen PY, Bakken S (2012). Review of health information technology usability study methodologies. J Am Med Inform Assoc.

